# *Ganoderma boninense*: general characteristics of pathogenicity and methods of control

**DOI:** 10.3389/fpls.2023.1156869

**Published:** 2023-07-10

**Authors:** Ying Wei Khoo, Khim Phin Chong

**Affiliations:** ^1^ State Key Laboratory for Biology of Plant Diseases and Insect Pests, Institute of Plant Protection, Chinese Academy of Agricultural Sciences, Beijing, China; ^2^ Faculty of Science and Natural Resources, Universiti Malaysia Sabah, Kota Kinabalu, Sabah, Malaysia

**Keywords:** control, diagnostics, *Ganoderma boninense*, oil palm, pathogenicity

## Abstract

*Ganoderma boninense* (*G. boninense*) is a soil-borne fungus threatening oil palm at the present. It causes basal stem rot disease on oil palm. Within six months, this fungus can cause an oil palm plantation to suffer a significant 43% economic loss. The high persistence and nature of spread of *G. boninense* in soil make control of the disease challenging. Therefore, controlling the pathogen requires a thorough understanding of the mechanisms that underlie pathogenicity as well as its interactions with host plants. In this paper, we present the general characteristics, the pathogenic mechanisms, and the host’s defensive system of *G. boninense*. We also review upcoming and most promising techniques for disease management that will have the least negative effects on the environment and natural resources.

## 1 Introduction

Basal stem rot (BSR), which has resulted in significant losses in oil palm plantations, is caused by *Ganoderma boninense* (*G. boninense*), a basidiomycete that is a member of the Polyporaceae family. However, other Ganoderma species have been linked to BSR (Chong et al., 2016). One of the major plantation crops in Malaysia and Indonesia is oil palm (*Elaeis guineensis* Jacq. Dura x Pisifera), and these two nations together account for 85 to 90% of global export (Kadarusman and Herabadi, 2018; Varkkey et al., 2018). According to studies by Stichnothe et al. (2014) and Kurnia et al. (2016), palm oil, which is widely recognised as one of the major sources of edible oil, also serves as a feedstock for oleochemicals and a precursor for biodiesel fuel. Nearly $24.66 billion in total export revenues from palm oil and palm oil products were reported for Malaysia (Parveez et al., 2022).

Despite the substantial export earnings from this product, oil palm plantations are hampered by the basal stem rot (BSR) disease, which severely reduces oil palm productivity and life cycle. For more than eight decades, BSR has been regarded as a serious threat to the oil palm sector. According to estimation by Olaniyi and Szulczyk (2020), *G. boninense* can wreak havoc on Malaysia’s oil palms by 2040, wiping out 860,610 ha of mature oil palm trees in the process. However, to date there is no published data on the exact damage areas caused by BSR. The most frequent species of fungus found to be the causal pathogen of BSR is *G. boninense* (Moncalvo, 2000; Susanto et al., 2005; Rees et al., 2007). In addition to mature oil palm trees, BSR also affects seedlings and younger plants, where the disease manifests quickly and severely (Susanto et al., 2005). BSR is characterised by the gradual decay of roots, bole and trunk tissue, which prevents water and nutrients from reaching the upper portion of adult oil palm trees. As a result, frond wilting, frond yellowing, un-opening of spear leaves, and eventually stand collapse occur (Chung, 2011). Unfortunately, oil palms infected with BSR are asymptomatic in the early stages of the infection, with the earliest symptom frequently being noticed on foliage when the infection has developed by 60–70% (Chong et al., 2017a). Young oil palm plants that exhibit disease symptoms typically die within 1 to 2 years, while mature trees only have a 3-to-5-year life expectancy (Corley and Tinker, 2003).

*G. boninense* management strategies continue to be difficult to implement despite extensive research efforts to control the diseases. In fact, interactions between the host plant, the pathogen, and the biotic and abiotic elements of the environment lead to diseases brought on by this soil pathogen are less explored. Therefore, our goals in this review are to (1) describe the general characteristics of *G. boninense*, (2) report the most recent findings regarding the mechanisms underlying pathogenicity as well as the interactions between the fungal pathogen and its host plants and/or other microorganisms, and (3) review the most effective current and upcoming control methods.

## 2 *Ganoderma boninense* general characteristics

*G. boninense*, a white rot fungus that degrade the lignin component of wood, belongs to the Basidiomycetes class under Ganodermataceae family (Paterson, 2007). Several *Ganoderma* spp. viz. *G. applanatum*, *G. boninense*, *G. chalceum*, *G. lucidum*, *G. miniatocinctum*, *G. pseudoferreu* and *G. tornatum* have been reported related to the oil palm basal stem rot in early investigations (Turner, 1981). However, *G. boninense* is the virulent species causing high incidence of BSR disease (Idris, 1999), and also a major species harmful to the oil palm (Moncalvo, 2000).

## 3 Morphological characteristics

The colonies of *G. boninense* were distinguished morphologically as having a white colour on the surface and dark pigment on the reverse. Under the dark condition, *G. boninense* cultures developed an undulating surface that buckled the agar. The ideal temperature for *G. boninense* growth is 30°C, and it can grow there at pH 3-8.5. Growth is severely hampered at 15°C and 35°C, and it is impossible above 40°C (Idris et al., 2000). After one to three weeks of incubation on rubber wood block (RWB), a white mycelium that later transformed into a small, white, button-like structure served as the initial indicator that a basidiomata of *G. boninense* had been artificially produced. When the bracket-like structures first emerged, they were typically white. However, as their length and width rapidly extended, their upper surfaces took on a variety of yellowish-brown colours with concentric zonations (Idris, 2009). Large, woody basidiocarps that are perennial are a characteristic of *G. boninense*. On the stems of infected palms, their fruiting bodies often develop into a fan-like pattern and contain double-walled, truncate spores with inner layers that range in colour from yellow to brown (Chong et al., 2017a).

## 4 Disease cycle

**Figure 1:**
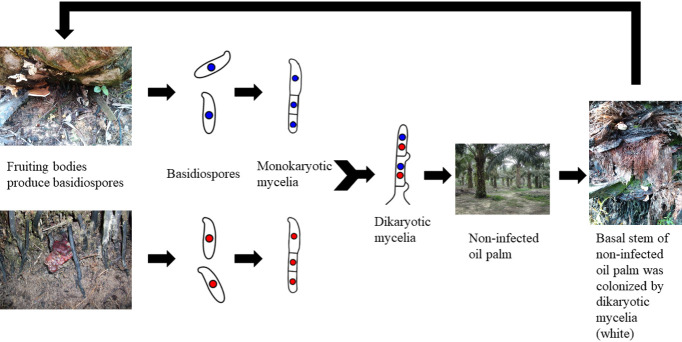
The disease cycle of *Ganoderma boninense*.

Contact between roots and a source of inoculum, such as stumps, trunks, and diseased oil palms, is related to the spread of BSR to healthy palms (Ariffin et al., 2000). Animals, insects, and the wind are all examples of vectors that can disperse the basidiospores that *G. boninense* fruiting bodies produce (Pilotti et al., 2003). Mycelia (monokaryotic form), which the basidiospore germinates into, is not pathogenic to the oil palm tree (Turner, 1965). The pathogenic dikaryotic mycelia are produced through mating between suitable mates of choice (monokaryon/dikaryon) (Freihorst et al., 2016). Then, infection occurred when the oil palm tree’s root and basal stem are colonised by the dikaryotic mycelia (Ariffin et al., 1996; Rees et al., 2009). In order to create a needle-like structure that will help them penetrate the host cells, dikaryotic mycelia will go through hyphae morphogenesis (Govender and Wong, 2017; Govender et al., 2020). Basidiocarps will form on the infected tree’s basal stem. As a result, the oil palm tree is infected with *G. boninense* (Rees et al., 2007). The disease cycle is summarised in **Figure 1**.

## 5 Diagnostics

Failure to identify symptoms and signs of infection at an early stage of disease development is a practical barrier to control of *G. boninense* disease in oil palm. The pathogen typically destroys a significant amount of a palm’s base before signs of infection appear (Darmono, 2000). In recent decades, many diagnostics tools have been developed and introduced to detect *G. boninense*. Milestones achieved in the development of diagnostic tools for detection of *G. boninense* are listed in **Table 1**.

**Table 1:** Achievement of milestones in the development of diagnostic tools for detection of *G. boninense*.

Year	Diagnostic tools	Feasibility	References
1996	*Ganoderma* selective medium (GSM)	GSM has the ability to selectively isolate the pathogen from afflicted tissue. Therefore, with the exception of *Ganoderma*, growing of fungus and bacteria is avoided in this procedure. However, because it requires a lot of labour and takes a long time, it cannot be used on a large scale. Other species of *Ganoderma* and some saprophytic fungi which is not a pathogen will also able to grow on this medium.	(Ariffin et al., 1996)
2006	Immunoassay	The MAbs produced against *G. boninense* were not specific because they displayed positive cross-reactivity results with other fungi examined.	(Sundram et al., 2006)
2007	Tomography system	Sonic Tomography image associated with a fuzzy inference is used to identify and recognize *Ganoderma* infection in oil palm stem. However, the model suffers from the variability of the points of view given by each expert.	(Su’ud et al., 2007)
2008	Hyperspectral Remote Sensing (HRS)	Oil palm leaves with and without *Ganoderma* infections differed in their spectral reflectance. Although the existence of *Ganoderma* infection in oil palm can be detected, early detection is still quite difficult. There is also confusion between the malnutrition versus diseased palms.	(Shafri and Anuar, 2008)
2009	Random Amplified Polymorphic DNA (RAPD)	Despite the *G. boninense* isolates’ high levels of homology, the RAPD study revealed variations. However, RAPD analysis still cannot be used to accurately identify *Ganoderma* spp.	(Zakaria et al., 2009)
2009	Ergosterol analysis	The development of *G. boninense* and the severity of the BSR illness in oil palm were directly correlated with ergosterol. As ergosterol is present in various types of fungi, not just *G. boninense*, this method may be able to quantify *Ganoderma* colonisation effectively without the presence of other fungi (Chong et al., 2014).	(Chong et al., 2009)
2009	Polymerase chain reaction (PCR)	PCR is regarded as a reliable reference technique for the early diagnosis of BSR disease. However, due to the need for expensive, time-consuming, and laborious stem collection and laboratory work, it cannot be used as a large-scale preparative experiment approach.	(Kandan et al., 2009)
2009	Intelligent electronic nose (e-nose) system	With artificial neural networks trained using the Levenberg-Marquardt technique, Cyranose 320 was used as the front-end sensors for decision-making. The smell of fungi was noticed. Both a laboratory and a plantation can use this technique.	(Markom, 2009)
2009	Microfocus X-ray Fluorescence (μXRF)	For both healthy and infected oil palms, element mapping and a specific line scan were carried out through the seventh leaf. However, it revealed some data overlap that has to be resolved in follow-up research.	(Meor Yusoff et al., 2009)
2010	Multiplex PCR	Multiplex PCR-DNA Kit can detect and identify the presence of *Ganoderma* species in oil palm, according to field studies.	(Idris et al., 2010)
2011	Ultrasonic	Density and ultrasonic characterizations have been used to find BSR-infected oil palm trunks. A high-power transducer should be taken into consideration to solve the issue of ultrasonic wave energy loss, particularly for large or young oil palm.	(Najmie et al., 2011)
2011	ITS—Restriction Fragment Length Polymorphism (RFLP)	ITS-PCR-RFLP have been demonstrated to help with genetic variation research among *Ganoderma*, but not down to the *G. boninense* strain.	(Nusaibah et al., 2011)
2011	Multispectral Remote Sensing (MRS)	Basal stem rot disease infections in oil palm fields may be located and estimated in detail and accurately using QuickBird imagery. Oil palms with BSR infection have lower infrared bands reflectance and higher Red-Green-Blue reflectance electromagnetic areas.	(Santoso et al., 2011)
2012	Airborne Imaging Spectrometer for Applications (AISA) sensor	AISA hyperspectral indices outperform both the currently used vegetation indices and red-edge approaches to detect *Ganoderma*-infected oil palm trees.	(Shafri et al., 2012)
2013	Infrared spectroscopy	Mid-infrared can be used to distinguish different BSR growth stages from oil palm leaves. To develop this strategy, particularly for early detection, more research is needed.	(Liaghat et al., 2013)
2013	DNA-microarray	Specifically for *G. boninense*, DNA-microarray is created., however, the processing of the samples and DNA extraction have been quite challenging.	(Dutse et al., 2013)
2014	Terrestrial Laser Scanning (TLS)	TLS can be used to distinguish between BSR and non-BSR, however it is limited in how severely it may be classified.	(Khairunniza-Bejo and Vong, 2014)
2014	Enzyme-linked immunosorbent assay-polyclonal antibody (ELISA-PAb)	ELISA-PAb outperforms the cultural-based method - *Ganoderma* selective medium (GSM) with an improvement of 18% at nursery trial. Use of oil palm roots and stems in a field study led to a 30% increase in sensitivity for ELISA-PAb detection when compared to GSM test. The identification of BSR illness brought on by *Ganoderma*, however, requires more specificity.	(Madihah et al., 2014)
2017	Artificial Neural Network (ANN) Spectral Analysis	ANN is only applicable for early-stage *Ganoderma* infection detection in oil palm	(Ahmadi et al., 2017)

The state of the art of CRISPR–Cas system has revolutionized nucleic acid detection. A recent study has adopted CRISPR-Cas12a as a diagnostic tool for detection of wheat fungi (Mu et al., 2022) and *Leptosphaeria maculans* (Lei et al., 2022). The advantages of robust nucleic acid detection make CRISPR-Cas12 widely used in plant disease and pest management (Karmakar et al., 2022). Based on those achievement, CRISPR-Cas12a is potentially to be developed as *Ganoderma* detection kit used in the field.

## 6 Genetic diversity

Amplification fragment length polymorphisms (AFLPs) has been combined with mitochodrial (mtDNA) profiling to provide a more comprehensive view of the molecular variation among the *Ganoderma* isolates (Rolph et al., 2000). After 7 years, microsatellite markers were used to study more about the genetic variation and population structure of *G. boninense* in Malaysia and Indonesia. The isolates of *G. boninense* from Peninsular Malaysia and Sumatra, Indonesia, showed a high level of genetic variation based on information from 11 microsatellite markers. The lack of genetic difference between *G. boninense* isolates from these two regions suggested that airborne spores may be a cause in the spread of *G. boninense* in these two regions (Mercière et al., 2017).

Based on the molecular characteristics of these *Ganoderma* species, the studies have revealed a high level of genetic variation among monokaryons of *G. boninense*, indicating that this species is genetically heterogeneous and may have developed from the same species with widespread genetic variation or from closely related species (Zakaria et al., 2005). This genetic heterogeneity may have been caused by different geographic locations or outcrossing of the isolates over generations or generation of new genetically distinct individuals through spore dispersal in the process of disease spread (Miller et al., 1999; Pilotti et al., 2003; Mohd Rakib et al., 2014). According to Pilotti (2005), *G. boninense* possesses a tetrapolar mating system, numerous alleles at both mating type loci, and is heterothallic, which encourages out-breeding within a population (Sanderson and Pilloti, 1997; Pilotti, 2005). Therefore, the extensive genetic variety exhibited in the *G. boninense* population on oil palm is caused by this potential to outcross.

*G. boninense* in Peninsular Malaysia, Sarawak, Malaysia, and Sumatra, Indonesia showed a significant degree of genetic variation when using 16 polymorphic microsatellite markers (Wong et al., 2021). In contrast to the low genetic variation reported in previous studies, due to *G. boninense*’s basidiospores’ outcrossing, high genetic variation was seen among isolates from the same population as well as between populations. *G. boninense* populations showed high levels of gene flow, but no genetic difference was found, suggesting that the BSR pathogen populations in three oil palm growing locations were homogeneous. Conclusively, Peninsular Malaysia, Sarawak, Malaysia, and Sumatra, Indonesia all have three admixed genetic clusters of the pathogen population (Wong et al., 2021). Conspecificity of *G. boninense* isolates from Indonesia, Malaysia, Papua New Guinea, and the Solomon Islands was shown based on mating studies, ITS sequences, and microsatellite genotyping (Pilotti et al., 2021). *G. boninense* isolates found in eight coastal sites in the Malaysian state of Sarawak showed low genetic variation. Using sequences from the internal transcribed spacer (ITS) region, low genetic variation was found in 117 isolates. The plantation was a first-generation planting that was formerly a secondary forest, which may be related to low disease pressure, which may account for the minimal genetic variation. First-generation plantation isolates of *G. boninense* are thought to be founder populations that eventually adapted to oil palm as the host (Midot et al., 2019).

## 7 Defense-related genes and proteins analysis

Numerous studies have uncovered defense-related genes involved in *G. boninense* infection of oil palm. Several genes related to oil palm defensive mechanisms were discovered by Tee et al. (2013), including genes associated to phytoalexin synthesis and genes associated to plant signal transduction. When the oil palm was infected by *G. boninense*, these genes were either upregulated to strengthen defence mechanisms or downregulated to weaken them. Ho et al. (2016) examined the transcriptomes of oil palm seedlings that had been infected and those that had not in order to deduce the molecular reactions of oil palm roots to the infection with *G. boninense*. Reactive oxygen species (ROS) such as hydrogen peroxide, which play a role in the oxidation of lipids and lignification of plant cells as well as acting as a signalling molecule inside the plant, are produced when enzymes like chitinases and glucanases are up-regulated (Ho et al., 2016).

Bahari et al. (2018) detected upregulated and downregulated gene sequences related to the change from the biotrophic to the necrotrophic phases of *G. boninense* in another transcriptome research. ROS elicitors, transcription factors EgERF113 and EgMYC2, defense-related genes (pathogenesis-related proteins, protease inhibitors, and chitinase), and defense-related genes were all differentially elevated (peroxidase and NADPH oxidase).

In another study, upon *G. boninense* infection, the regulation of a number of oil palm genes changes. Receptor-like kinases and proteins (RLKs and RLPs), which are important in recognising the pathogen, are extensively produced during the early stages of infection (Rosli et al., 2018).

In 2021, through RNA-sequencing (RNA-seq) transcriptomic libraries of oil palm roots infected with *G. boninense*, seven differentially expressed genes (DEGs) have been detected. These seven DEGs are anthocyanidin synthase, chalcone synthase-like, transcription factor 1B-like, leucoanthocyanidin reductase-like, mannose-specific lectin-like, putative senescence-associated protein, thaumatin-like protein. During *G. boninense* infection, seedlings and mature plants consistently displayed upregulation of seven putative defense-related DEGs. These seven genes may be developed into biomarkers for the early detection of BSR in oil palm (Mohd Zuhar et al., 2021).

## 8 Pathogenesis of *Ganoderma boninense*

An earlier study revealed that various methods, viz. debris, root contact and basidiospores, are used by *Ganoderma* sp. for colonisation in the oil palm plantation (Turner, 1965; Pilotti et al., 2003; Pilotti et al., 2018; Govender et al., 2020). Turner (1965) showed that *G. boninense* can continue to spread via debris from colonised oil palm trees after new plants are planted. Besides, mycelia can colonise on healthy, damaged or dead roots (Miller et al., 1999; Govender et al., 2020). The continuous grow of the oil palm roots to the first four planting rows lead to the root-to-root contact between palms, which facilitates the spread of pathogen (Soepena et al., 2000). The increasing number of patches caused by BSR infection over time support the theory stated the roots are the main source of inoculum in the field (Miller et al., 1999). Infective dikaryotic mycelia continue to form when germinated basidiospores mating with the mycelia (Pilotti et al., 2003; Pilotti et al., 2018).

## 9 Oil palm – *Ganoderma boninense* interaction

The process of *G. boninense* colonising the root surface is crucial for the pathogen to later penetrate between host cells and finally cause host harm (Rees et al., 2009). The root cortex becomes colonised by *G. boninense* during the biotrophic phase’s initial infection, with many hyphae filling the host cells’ completely intact cell walls (Rees et al., 2009). The epidermal surface of the oil palm root tissue served as the entry point for *G. boninense*’s infection, which then led to the xylem vessels (Alexander et al., 2017a). Pathogen-associated molecular patterns (PAMPs) from *G. boninense* may bind to pattern recognition receptors (PRRs) that are extracellular or located at the plasma membrane to begin the process of pathogen recognition by oil palm (Ho et al., 2016). In oil palm-*Ganoderma* pathosystems, chitin, glucans, and ergosterol, components of the fungal cell wall or cell membrane, have been identified as PAMPs (Ho et al., 2016).

After establishing robust biotrophic growth and defeating the host defenses, the fungus enters a nectrotropic phase, which is characterised by significant cell wall lysis and the death and maceration of host tissues (Whalen, 2005; Alexander et al., 2017a). As a result of *G. boninense* infection, several oil palm chitinases and glucanases were in fact up-regulated in oil palm roots. The fungal cell wall may be broken down, loosen, and soften by these cell wall degrading enzymes (CWDEs) viz. cellulase, laccase, manganese peroxidase and polygalacturonase, releasing chitins and glucans (Breton et al., 2009). As an alternative, ergosterol, which has been implicated in pathogen virulence (Doehlemann et al., 2008), may similarly induce PAMP-triggered immunity (PTI) in oil palm. The oxidative degradation of lignin and the white rot status attributed to *G. boninense* may be detected by the production of melanized mycelium (Adaskaveg et al., 1990; Paterson, 2007). At last, the tissues of the oil palm roots lost their integrity and seemed to be a decaying mass covered in thick fungus mycelium. As the roots rot, the pathogens act as a source of inoculum and survive for a long time in the soil (Hasan and Turner, 1994). Once this inoculum found an appropriate host with a favourable environment, it began to actively spread (Chong et al., 2017c).

## 10 Management strategies

### 10.1 Genetic resistance

To date, there is no vertical resistance to BSR that inhibits or limits infection (Rozlianah et al., 2015). Instead, there are the horizontal and partial resistance that do not limit infection but reduces or compensates the damages, and as a result, the effects on the fitness of oil palm (Idris et al., 2004b; Durand-Gasselin et al., 2005; Breton et al., 2006; Wening et al., 2016). Breeding program has used South American *E. oleifera* and African *E. guineensis* species as genetic resources (Soh et al., 2017) because different BSR susceptibilities were found in these two species (de Franqueville et al., 2001; Durand-Gasselin et al., 2005).

In the past few decades, Ariffin et al. (1999) has discovered the tolerant progeny of oil palm to BSR viz. Cameroon x Cameroon, Cameroon x AVROS and Nigeria x Nigeria. Then, in 2004, Idris et al. (2004b) has discovered the sources of tolerance genes in Zaire x Cameroon bred, and the sources of partial resistance gene in Congo x Cameroon bred. A year later, the source of partial resistance from the Magenot line of *E. oleifera*. *E. guineensis* of Deli origin from Malaysia and Indonesia was found to be more susceptible to BSR than African material in North Sumatra, Indonesia (Durand-Gasselin et al., 2005). In the study of Chong et al. (2012), AVROS has been reported less susceptible to BSR compared to Calabar and Ekona. Two years later, Turnbull et al. (2014) has identified the potential resistance sources in La Mé A x Deli Dabou B and Yangambi IRHO A x Deli Socfindo. At the same time, ‘D x P Socfindo Moderate Gano Resistant’ which is a *G. boninense* partial resistance material has been released by PT Socfindo (Tan et al., 2018). In 2016, another partial resistance material namely ‘Yangambi *Ganoderma*-Tolerant 1’ has been released by FELDA Global Ventures (Tan et al., 2018). Despite the breeding program’s promise, it is unlikely to quickly produce tolerant or resistant *Ganoderma* planting materials.

Oil palms that are tolerant or resistant to BSR may offer a long-term approach of BSR control. However, the lack of quick and efficient screening procedures makes it difficult to generate resistant or tolerant varieties (Soh, 2017). Recently, the study of *Ganoderma* resistance in oil palm have been explored at the genetic level. The introduction of QTL (Quantitative Trait Loci) mapping in breeding program has efficiently and flexibly generated valuable data for the identification of resistance loci (Tisné et al., 2017). There were found to be four *Ganoderma* resistance loci, of which two affected the occurrence of BSR symptoms and two affected the rate at which oil palm trees died (Tisné et al., 2017). The ability to combine known favorable genomic regions, such as those for disease and yield related traits, will help with multicriteria improvement of oil palm by preventing unintentional unfavorable selection on the others. This will be made possible by the localization of *Ganoderma* resistance loci (Tisné et al., 2017).

Through the rapid multiplication of plantlets with uniformity and desired features, tissue culturing outperforms conventional breeding (Ngando-Ebongue et al., 2012). Lignin has been associated with the protection of oil palm lignin’s cell wall structure and function, which play a key part in protecting plants. Increase the amount of lignin of oil palm has been considered using tissue culture technique. However, the palm tissue culture is a long process and time is required for parameters of the transformation to be optimized (Sahebi et al., 2015).

Increasing accumulated Si in oil palm using genetical manipulation methods has been proposed. The TEM images demonstrate that intracellular regions of the root and leaf cells create a protective layer of cellulose-Si membrane when oil palm is subjected to Si treatment. The accessibility of the fungal mycelium to the middle lamella component of the cell wall is thought to be affected by the Si deposition in the intercellular regions of the cell (Sahebi et al., 2015).

### 10.2 Chemical control

#### 10.2.1 Fertilizers

Mineral fertilizers play a vital role in enhancing plant and soil health, and microbial activity (Singh and Ryan, 2015). Rahman and Othman (2020) have applied calcium carbonate to oil palm seedlings, and the best soil pH for suppressing BSR was pH 6. Integration of mineral fertilizers with other chemicals or living organisms have been performed in *Ganoderma* research. Purba et al. (1994) has demonstrated the integration of sulphur, cover crops and tridemorph fungicide has reduce the incidence of BSR after 5 years of application. Calcium nitrate and the biological control agent (BCA), *Trichoderma* sp., reduced illness, according to Sariah and Zakaria (2000) and Naher et al. (2018). Silicon oxide, potassium silicate, calcium silicate, sodium silicate, and sodium meta-silicate, according to Najihah et al. (2015), reduced the severity of BSR in oil palm seedlings. Silicon has positive impacts on growth, yield, and disease resistance in plants (Wang et al., 2017). Continuous calcium, copper, and SA supplementation may be essential for improving disease resistance in oil palm (Rahamah Bivi et al., 2016), and greater lignin content was also noticeable, suggesting a possible mechanism for infection resistance.

#### 10.2.2 Fungicides

Oil palm plants infected with BSR have been treated with fungicides via trunk injection, soil drenching, combo treatments using both methods, pressure injection, soil injection, and soil fumigation (Ariffin and Idris, 1991; Ariffin et al., 2000; Idris et al., 2002; Idris et al., 2004a). Initially, diseased palms have been painted with carbolineum (fungicidal paste) after discarding basidiomata (Turner, 1981). Issue with pollution that poses a health risk persists after using carbolineum (Osterloh and Nusser, 2014).

Later, *Ganoderma* infected palms in the field has been reported to be highly inhibited by organic mercury formulations after trunk injection, however the residue issue made them unusable for commercial application (Turner, 1981). In 1990, Khairudin (1990) found out that injecting systemic fungicides like fusilazol, hexaconazole, cyproconazole, flutriafol, triadimenol, tridemorph, and oxycardoxin slowed the spread of BSR. Application of hexaconazole via pressure injection, trunk injection, soil drenching, and soil injection, into diseased oil palms reduced the spread of BSR in the infected oil palms (Idris et al., 2002; Idris et al., 2004a). Pressure injection has been used to accurately apply the hexaconazole to the infected area. Idris et al. (2004a) reported that after 5 years, 70% of treated oil palms produced fruit bunches. In Jee and Chong (2015) study, despite being given hexaconazole, ergosterol was found in the diseased tissues both before and after the treatments. But, at least, hexaconazole treatment has prolonged the period of productivity in *Ganoderma*-infected palms (Idris et al., 2004a).

Besides, Ariffin and Idris (1991) have reported that the formation of fruiting bodies might be inhibited by injecting the fumigant fungicide dazomet into the stem lesion which further reduced the spread of BSR. Idris and Maizatul (2012a) also reported that *Ganoderma* inoculum in infected stumps at three years after dazomet treatment has been eradicated. Later, Idris and Maizatul (2012b) observed that dazomet increased oil palm productivity by reducing the spread of BSR by eradicating *Ganoderma* inoculum.

The effectiveness of pyraclostrobin, a broad-spectrum fungicide with curative, preventative, and long-lasting characteristics, in preventing the growth of *G. boninense* has been assessed. Pyraclostrobin proved successful in reducing BSR in oil palm seedlings while also having beneficial physiological benefits on seedlings, such as increased height, bole diameter, root mass, photosynthetic rate, PSII quantum efficiency, and relative leaf chlorophyll content (Said et al., 2019). However, this fungicide’s efficacy on mature oil palms has not yet been investigated.

Nanomaterials are being applied as carrier for fungicides delivery. Hexaconazole-zinc and Dazomet-zinc/aluminum layered double hydroxide nanocomposite has performed better than pure dazomet and hexaconazole as a fungicide and a micro-nutrient (Mustafa et al., 2018a; Mustafa et al., 2018b). Hexaconazole-zinc/aluminum layered double hydroxide nanocomposite exhibits superior growth inhibition against *G. boninense* than pure hexaconazole (Mustafa et al., 2018a). Hexaconazole and dazomet were successfully delivered to *G. boninense* cells using a nanodelivery system that used chitosan nanoparticles as the carrier for the fungicides. Low phytotoxicity and high activity against the pathogen were displayed by the nanodelivery system (Maluin et al., 2019a; Maluin et al., 2019b).

Despite the fact that some effective fungicides are available, utilizing fungicides in control approaches might cause environmental contamination and the emergence of fungicide resistance (Shcherbakova et al., 2020). For the effective control of *Ganoderma*, cultural control techniques such as field hygiene or the prevention of pest immigration are essential. Since it is hard to control all pest species using biological control, it is crucial for commercial application to combine the use of counterpart of *Ganoderma* with other control strategies, such as cultural control and the use of chemicals.

### 10.3 Cultural practices

In established oil palm plantations, it is frequently advised to manage BSR through good cultural practises. To stop the spread of disease, cultural methods often involve eliminating and decreasing the pathogen inoculums to provide oil palm with short-term relief. These include clean clearance, sanitation, soil mounding, surgery, trenching, and windrowing.

#### 10.3.1 Clean clearance

Excision and removal of all leftover fragments from an infected palm area is known as “clean clearance,” and it involves digging holes that are 1.5 m^2^ and 60 cm deep from both untreated vacant points and infected palm points (Singh, 1990). Despite the practice of clean clearance during the replanting of second- and third-generation palms, residual root pieces can play a significant role in the emergence of BSR. The ability of the root fragments to form *G. boninense* basidiomata, which are occasionally found on their cut ends, indicates that they still possess adequate inoculum potential to spread disease (Ariffin et al., 2000). Normally, all of the still-present fragments of the infected palm are brought to the surface and either removed or burned. However, this strategy is expensive, and many nations that produce palm oil restrict open burning. As a result, it is usual practise to shred all palm pieces, which can then be either dispersed across the entire field or stacked in rows and covered with a legume cover crop to speed up decomposition (Chong et al., 2017b). Although these procedures typically reduce the occurrence of BSR in newly planted oil palms, infection can nonetheless gradually take place due to debris left in the soil (Singh, 1991).

#### 10.3.2 Sanitation

When an appropriate disease inoculum is present, diseased stumps are the tissues that are most contagious. The oil palm will be attacked by the *Ganoderma* inoculums in these stumps if there is a contact. Therefore, it is crucial to maintain field sanitation and the removal of diseased tissues in order to keep the plantation free of pathogen sources (Chong et al., 2017b). In this sanitation operation, a sizable hole measuring 2 m x 2 m x 1 m is dug (Idris et al., 2004a). The infected materials are removed, crushed into little pieces, and placed in the interrow spaces to decompose naturally. In the majority of sizable plantations, this technique is frequently used while replanting (Chung, 2012). Prior to replanting, sanitation had greatly reduced the prevalence of BSR on the replanted palms by removing soil and old stumps, ploughing, and rotovating. Ten years after replanting, the BSR incidence was lower in places with sanitation than it was in areas without sanitation, at less than 1% as opposed to more than 13% (Ahmad et al., 2012).

#### 10.3.3 Soil mounding

By preventing weakened boles from being blown over by wind, soil mounding, which involves gathering dirt from nearby regions and piling it up to a height of 0.75 m up and 1 m radius wide around the trunk, inhibits the progress of BSR in infected trees (Ho and Khairudin, 1997; Susanto et al., 2009). However, the spread of *Ganoderma* was not slowed down by this strategy, but it did aid to extend the economic life of yielding palms that were afflicted by BSR (Chong et al., 2017b).

#### 10.3.4 Surgery

In some plantations, surgery is performed by mechanically removing infected tissues using a backhoe blade (Singh, 1991) or manually removing them with a hand-held chisel (Turner, 1981). Following surgery, palms have shown improved survival and production (Hasan and Turner, 1994). However, surgery frequently failed because of late identification, a huge disease lesion, and a lesion that extended below ground, including infected root masses, leading treated palms to fall at the end (Chung, 2011).

#### 10.3.5 Trenching

Digging trenches to prevent palm-to-palm contact is another cultural practise that has been adopted. Compared to windrowing and open clearing, it might be a preferable choice (Chong et al., 2017b). Wakefield (1920) recommended digging trenches around infected palm trees as a control measure to stop the pathogen from spreading to nearby healthy trees. Oil palm on peat field trials in Indonesia have demonstrated that the establishment of isolation trenches measuring 4 x 4 x 0.75 m deep can effectively reduce the transmission of BSR infection to nearby healthy oil palms (Lim and Udin, 2010). According to a field trial by Lim and Udin (2010), isolation of trenches around old stumps is successful in preventing BSR infection for oil palm planted between the old stands for up to 14 years. However, trenches have not been effective because they were either not maintained or their depths were insufficient to stop roots from passing underneath (Turner, 1981; Ariffin et al., 2000). This control measure must also be regularly maintained because erosion of the trench edges will cause partial filling (Ariffin et al., 2000). In general, this measure is not frequently used (Chong et al., 2017b). Surgery and trenching are not widely practiced nonetheless being recorded earlier.

#### 10.3.6 Windrowing

Oil palm trunks that have been felled and root tissues that have been removed are put along the previous rows using the windrowing technique. This method has proven to be virtually as effective at limiting losses in the cultivated crop as clean clearing yet involving less labour (Chong et al., 2017b). But according to a comparative study by Hashim (1991), windrow treatment (27.3-17.6%) and clean clearing (from 27.3% in the prior stand to 14% in the replanted stand after 15 years) were the most successful in reducing the incidence of BSR.

### 10.4 Biological control

Biological control agents (BCAs) are becoming more popular as a potential replacement for chemical fungicides for controlling BSR in oil palm fields (Sujarit et al., 2020). Many BCAs have been studied to control *Ganoderma* in the last few decades. Most of the BCAs have been conducted *in vitro* as reviewed by Supramani et al. (2022). In *in vitro* studies, *Ganoderma boninense* has been inhibited to some extent by some BCAs viz. actinomycetes, arbuscular mycorrhizal fungi, *Bacillus* spp., *Diaporthe* spp., mycoparasite, *Penicillium* spp., *Streptomyces* spp. and *Trichoderma* spp. (Bakhtiar et al., 2010; Sundram et al., 2011; Azizah et al., 2015; Goh et al., 2015; Chow et al., 2017; Lim et al., 2018; Chow et al., 2019; Sujarit et al., 2020). Inhibition happened attributed to some potential antimicrobials compound such as eudistomin I, Gly-Met-OH, halstoctacosanolide A, lovastatin, naringenin, N-acetyl-leu-leu-tyr-amide, N-methyl-a-aminoisobutyric acid, penipanoid A, phenazine and phenazine-1-carboxylic acid, phenylethyl alcohol, phenyl alkenoic acids, pyrene-1,6-dione, 4-O-8’,5”-5’-dehydrotriferulic acid, 7-O-beta-D-glucoside, 12-deoxyaklanonic acid, and 12-oxo-10Z-dodecenoic acid (Angel et al., 2016; Alexander et al., 2017b; Lee et al., 2018; Ahmad et al., 2020; Sujarit et al., 2020). However, *in-vitro* studies conducted in nursery and field trials are required to validate the efficiency of those BCAs. Some examples of BCAs which have showed their efficiency in BSR control in *in vivo* are listed in **Table 2**.

**Table 2:** List of biological control agents (BCAs) in *in vivo* studies.

BCAs	Oil palm varieties	Effect	References
*Trichoderma harzianum*, *Glomus etunicatum*	Dura x Pisifera	In oil palm infected with *T. harzianum* and *G. etunicatum*, the density of the soil microbial population rose.	(Alizadeh et al., 2013)
*Aspergillus* sp., *Bacillus* spp., *Lactobacillus*, *Nattobacillus*, *Pseudomonas* spp., *Saccharomyces* *cerevisiae*, *Trichoderma* spp.	AVROS (Dura x Pisifera)	A field trial revealed that TR1 (*Bacillus* spp. & *Trichoderma* spp.) and TR3 (*Lactobacillus*, *Nattobacillus* and *Saccharomyces cerevisiae*) significantly reduced the disease index (DI) to 12% and 24%, respectively, and the amount of ergosterol in trunk tissues to 0.663 g g-1 and 1.817 g g-1. The BSR disease was successfully controlled by TR1, TR2 (*Bacillus* spp., *Pseudomonas* spp. & *Aspergillus* sp.), and TR3 in both the nursery and the field.	(Alexander and Chong, 2014a)
*Bacillus* spp., *Trichoderma* spp	No stated	The soil’s beneficial microbe population grew as a result of the addition of BCAs viz. *Bacillus* spp. (TR1) and *Trichoderma* spp (TR2), which can be used in conjunction with BCAs to control BSR.	(Alexander and Chong, 2014b)
*Bacillus* spp., *Trichoderma* spp.	No stated	The antimicrobial compounds N-methyl-a-aminoisobutyric acid, pyrene-1,6-dione, 12-deoxyaklanonic acid, halstoctacosanolide A, N-acetyl-leu-leu-tyr-amide, and 12-oxo-10Z-do 4-O-8’,5″-5’-dehydrotriferulic acid found in TR1 (*Bacillus* spp. and *Trichoderma* spp.) that can inhibit *G. boninense*.	(Alexander et al., 2017b)
*Trichoderma harzianum* *Bacillus cereus*	GH500 (Dura × Pisifera)	With a reduction of 94.75%, *B. cereus* single applications outperformed *T. harzianum* (78.98%) and *T. harzianum* and *B. cereus* combined applications as the most effective treatments for controlling *Ganoderma* disease of oil palm.	(Nusaibah et al., 2017)
*Diaporthe phaseolorum* WAA02, *Trichoderma asperellum* T2, *Penicillium citrinum* BTF08)	Tissue-cultured oil palm ramets	Induction of β-1-3-glucanase (*EgGLC*), phenylalanine ammonia-lyase (*PAL*) and nitrate reductase (*EgNR*) by Endophytic BCAs were possibly responsible for the defense responses.	(Chow et al., 2018)
*Trichoderma asperellum* LF11 *Diaporthe miriciae* LF9	No stated	Oil palm seedlings with *D. miriciae* LF9 infection had lower disease incidence (DI) in non-metal (33%) and multi-metal (67%) treated soils, indicating that the seedlings were more resistant to infection. *T. asperellum* LF11 performed less well than *D. miriciae LF9*.	(Sim et al., 2019)
*Trichoderma virens*	Dura x Pisifera	The isolate’s hexane extract inhibited *G. boninense* at a rate of 62.60%. The enzyme activity of peroxidase, polyphenol oxidase, superoxide dismutase, and phenylalaninelyase were noticeably increased in the leaves of oil palm seedlings following treatment with *T. virens* isolates via plant roots. *T. virens* can function as a biofungicide.	(Paudzai et al., 2019)
*Trichoderma* *asperellum*, *Trichoderma virens*	No stated	When compared to the control treatment, the illness was reduced by 38.59% and 50.87%, respectively, in treatments employing *T. virens* T29-palm press fibre (PPF) surface mulch (T5) and *T. asperellum* T9-PPF surface mulch (T4).	(Sundram, 2013)
*Trichoderma* *asperellum*, *Trichoderma* *harzianum*, *Trichoderma virens*	No stated	The combination of Trichoderma spp. decreased the disease by 83.03% and 89.16%, respectively, in foliar and bole symptoms. Treatment for BSR disease results in significant alterations in peroxidase, polyphenol oxidase, and phenolic content. The link between oil palm metabolism and defense mechanisms may be the subject of future study.	(Musa et al., 2018)
*Streptomyces sanglieri*	No stated	It has been discovered that *S. sanglieri* produces the antifungals cycloheximide and actiphenol, both of which may be effective against *G. boninense*.	(Nur Azura et al., 2016)
*Scagelonema parasiticum*	Dumpy Yangambi Avros (Dura x Pisifera)	Cellulolytic and xylanolytic antifungal activity was displayed by *Scagelonema parasiticum*. *S. parasiticum* has a mycoparasitic growth pattern and produces fluorescent pigments and/or metabolites, making it a potential biocontrol agent for *G. boninense*.	(Goh et al., 2016)

BCAs indicated as biological control agents.

BSR indicated as basal stem rot.

Recently, virus and viroid-like RNAs have been detected in fungi (Dong et al., 2022; Liu et al., 2022). A mycovirus called SlAV1 from the plant pathogen *Stemphylium lycopersici* that causes pigmentation loss and hypovirulence by preventing the fungus from biosynthesising the phytotoxin Altersolanol A. The pathogen becomes a BCA and improves plant resistance to virulent strains when its genome is integrated and a crucial SlAV1 gene is expressed in the fungal host (Liu et al., 2022). Besides, *Botryosphaeria dothidea* RNAs (BdcRNAs), a viroid-like RNAs similar to those of viroids have exhibited pathogenic effects on hosts (Dong et al., 2022). Their hypovirulence have conferred them the ability as potential BCAs to control fungi. Considering the current aforementioned findings, there is a possibility that *G. boninense* might have mycovirus or viroid-like RNA to control it and improve oil palm resistance, but yet to be discovered in the future. To date, only Coconut cadang-cadang viroid (CCCVd) has been reported to cause orange spotting on fronds of oil palm (Vadamalai et al., 2009). This finding may raise the question of whether CCCVd-infected oil palm would have better resistance against *G. boninense*. If yes, what is the mechanism behind this viroid-fungi-host relationship? Peptidases are secreted by both pathogenic fungi to break down a range of (poly)peptides in their environment (Amselem et al., 2011). In the finding of Dong et al. (2022), for example, BdcRNA 1 transfectants have down regulated the DEGs related to peptidase activities, and thus attenuated the fungi virulence. This finding has provided the novel insight on understanding the relationship of mycoviroid and fungi in their host.

### 10.5 Elicitors of plant defense

Elicitors are compounds that elicit various plant defensive responses (Thakur and Sohal, 2013). Elicitors have been shown to induce hypersensitive response (HR) and systemic acquired resistance (SAR) in plants to fend off infections from bacteria, fungi, and viruses (Zhang et al., 2004; Pawlowski et al., 2016). The effectiveness of elicitors amino acid (AA), benzoic acid (BZA), caffeic acid (CA), chitosan, fulvic acid (FA), humic acid (HA), jasmonic acid (JA), salicylic acid (SALA), syringic acid (SA) and 4-hydroxybenzoic acid (4-HBA) have been examined on *G. boninense in-vitro* or *in-vivo* (Ommelna et al., 2012; Jee and Chong, 2015; Rahamah Bivi et al., 2016; Chong et al., 2017b; Ong et al., 2018; Surendran et al., 2018). In this subsection, elicitors are reviewed and recommended (**Table 3**).

**Table 3:** Elicitors and their amount and effect on tested variety with recommendations.

Elicitor	Amount or Concentration	Tested variety/type	Effect	Recommendation	Reference
BZA	15mM	Dura X Pisifera (DXP)	Internal and exterior oil palm seedlings treated with 15mM benzoic acid showed no signs of illness up until the 8 month after inoculation.	Field tests are required.	(Surendran et al., 2018)
BZA	–	–	*G. boninense* did not develop on the plates that were exposed to benzoic acid concentrations more than 5.00 mM.	Glasshouse and field tests are required.	(Fernanda et al., 2021)
Chitosan	0.5% (w/v)	AVROS	Chitosan at the lowest concentration of 0.5% (w/v) significantly reduced the amount of fungal sterol and resulted in the lowest percentage mean disease severity.	Field tests are required.	(Ommelna et al., 2012)
SALA	150 ppm	–	Suppression of mycelial growth on PDA until 8 day-after-inoculation	Field tests are required.	(Ong et al., 2018)
Calcium chloride/copper-EDTA/SALA	500/50/50 ppm	–	The best disease control (5.0%) is noticed until 8 month-after-inoculation	Field tests are required.	(Rahamah Bivi et al., 2016)
CA/SA/4-HBA	4g each	–	Concentration of *G. boninense* ergosterol is reduced to minimum (-18.6888 μg g^-1^)	The combination of these phenolics is encouraging to be produced as products	(Jee and Chong, 2015)
FA/HA/MBCA	–	–	Concentration of *G. boninense* ergosterol is reduced to minimum (-1.81 μg g^-1^)	Field tests are required. These combinations are encouraging to be produced as products	(Chong et al., 2017b)
Chitin/AA/MBCA	–	–	The Infection is reduced down to 10% with 2.29 μg g^-1^ of ergosterol content	Very encouraging. However, more work on fastens the efficiency of treatment is required	(Chong et al., 2017b)

AA indicated as amino acid.

BZA indicated as benzoic acid.

CA indicated as caffeic acid.

EDTA indicated as ethylenediaminetetraacetate.

FA indicated as fulvic acid.

MBCA indicated as multiple biological control agents.

SALA indicated as salicylic acid.

4-HBA indicated as 4-hydroxybenzoic acid.

It has been noted that chitosan, a natural polymer can be found in the shells of insects, fungus, and crustaceans have protected various crops from harmful fungus by triggering their natural defense response (Bhaskarareddy et al., 1999; Hadwiger, 1999; Takeshi et al., 2000). In the study of effectiveness of chitosan against *G. boninense*, the findings implied the fungal sterol of *G. boninense* in oil-palm root was suppressed and the lowest percentage mean disease severity was recorded when treated with chitosan at a minimum concentration of 0.5% (w/v). Result also showed that there was no discernible difference between any of the chitosan concentrations for bole tissue infection as no infection was found. Compared to seedlings that were pre-treated with chitosan and later infected with *G. boninense* at the same concentration, *Ganoderma*-infected seedlings that were treated with chitosan (0.1%, w/v) displayed slightly less severe illness (Ommelna et al., 2012).

Salicylic acid (SALA), a naturally occurring plant hormone, gained interest after it was discovered that it can increase plants’ tolerance to abiotic stress and disease resistance (Alonso-Ramírez et al., 2009; Gautam and Singh, 2009; Pieterse et al., 2009). Effectiveness of SALA has been examined *in vitro* on *G. boninense* growing in potato dextrose agar (PDA). The finding indicated that mycelial growth recovery was seen starting on 9 day-after-inoculation for SALA levels of 150 ppm and above (Ong et al., 2018). In a glasshouse experiment, Rahamah Bivi et al. (2016) shown that SA decreased disease incidence and severity brought on by *G. boninense* in oil palms. The findings are in contrast to those of Ho et al. (2020), who discovered that the disease symptoms of infected oil palm seedlings treated with SALA and the untreated oil palm seedlings were not significantly different. However, Mohan et al. (2022) showed that during the biotrophic phase of *Ganoderma* infection, SALA was involved in the early defense response from infected oil palm.

Jasmonic acid (JA) has become important signaling molecules linked to stress resistance and plant development (Ghorbel et al., 2021). JA has been reported to promote the growth of *G. boninense* in PDA starting on 7 day-after-inoculation regardless of different concentrations (Ong et al., 2018). Ho et al. (2020), who demonstrated that the illness symptoms of infected oil palm seedlings treated with JA and the untreated oil palm seedlings were not substantially different, provide additional support for the findings (Ong et al., 2018). The results are contrast to the current study (Mohan et al., 2022). During the necrotrophic phase of *Ganoderma* infection, JA mediated the defensive response from the root lesions of *Ganoderma*-infected oil palm root tissues.

Benzoic acid (BZA) plays a crucial role in the composition of defensive chemicals (Widhalm and Dudareva, 2015). Benzoic acid at a dose of 15 mM has been shown by Surendran et al. (2018) to be an effective control against BSR disease in oil palm. More than 80% of the growth of *G. boninense* was inhibited by treatment with benzoic acid. The mycelia of *G. boninense* were severely structurally damaged by the benzoic acid treatment (5.00 mM). *G. boninense* mycelia that had been treated had distinct holes and ruptures and were thinner, less dense, deformed, and shriveled. However, demonstration of field and glasshouse studies are scarce (Fernanda et al., 2021).

Besides benzoic acid, other phenolic acids such as caffeic acid (CA), syringic acid (SA) and 4-hydroxybenzoic acid (4-HBA) have been applied to control *G. boninense* in the estate. The combination of 4 g of each phenolic acid is the concentration that suppresses *G. boninense* the most effectively, leading to the greatest decrease in *Ganoderma* ergosterol in infected tissues (Jee and Chong, 2015).

Fulvic acid (FA) is a type of plant growth regulator that enhance plant growth and plant stress resistance (Peiris et al., 2011; Sun et al., 2020). Humic acid (HA) is an organic product that control several biochemical effects, such as cell membrane permeability, photosynthetic rate, cell elongation, and increase water use efficiency (Zhang et al., 2013). Combination of FA and HA with multiple biological control agents (MBCA) have been demonstrated to control *G. boninense*. By adding HAs and FAs, the soil becomes naturally equipped to fend off many fungi that are capable of causing soil-borne diseases (Chong et al., 2017b). Oil palm seedlings in nursery were used to assess the effects of adding multi-BCAs, chitin, and AA.

### 10.6 Oil palm secreted-plant metabolite

In response to a number of environmental variables, plants generate a numerous of primary and secondary metabolites. Certain metabolites have significant roles in plants’ defensive systems against fungi, bacteria, and other pests, according to secondary metabolite profiling.

These metabolites play a role in defensive processes during interactions between plants and pathogens (Bennett and Wallsgrove, 1994; Pedras and Ahiahonu, 2005; Hasegawa et al., 2010). As metabolites of plant defence against infection with *G. boninense*, several steroid molecules have been discovered. The crude root extracts of infected tolerant and susceptible oil palm progenies included the three primary steroids sitosterol, -sitosterol, and stigmasterol. Stigmast-5-en-3-ol, campesterol, cholestan, and an ergosterol (- and -tocopherol) were also found (Nusaibah et al., 2011). Other metabolites related to the early defence mechanism of the oil palm included lipids like fucosterol, betasitosterol, gamma-sitosterol, palmitic acid, and linoleic acid, antioxidants like -tocopherol and -tocopherol as well as heterocyclic aromatic organic compounds like pyridine, benzo[h]quinoline, and pyrimidine. These metabolites were observed in the root extract of infected samples, and it was discovered that resistant oil palm progeny had higher quantities of these metabolites than susceptible oil palm progeny (Nusaibah et al., 2011; Nusaibah et al., 2016). Based on the current findings, the resistant progeny increased the number and concentration of metabolites in the roots that had antifungal properties during the early stages of *G. boninense* infection. Because of this, the resistant progeny can exhibit greater levels of resistance than the susceptible progeny (Nusaibah et al., 2016). These biomarkers were found in *G. boninense*-infected oil palm, which indicates that they are part of the oil palm’s defence mechanism against the pathogen. Therefore, an early detection could help to extend the palm’s economic life (Nusaibah et al., 2016).

### 10.7 Plant and alga extracts

Plant and alga extracts have been studied to inhibit the growth of *G. boninense in vitro.* Plant extracts such as carboxylic acid, ester, fatty acid ester, phenol and steroid caused the inhibition growth of *G. boninense in vitro* (Tay and Chong, 2016). According to Abdul Aziz et al. (2019), extracts from algae *Caulerpa racemosa*, *C. racemosa* var. lamouroxii and *Halimeda macrophysa* have been reported to exhibited antifungal activities on *G. boninense* at the lowest concentration tested. Further research can be conducted to apply these bioactive compounds to confer resistance to oil palm in the field.

### 10.8 Extracts of bee-produced products

Phenolic compounds such as Gallic acid (GA) and Thymol (THY) have been extracted from bee-produced products. These compounds have showed their significant inhibition of the mycelial growth of *G. boninense*. Gallic acid at a dose of 8 mg/mL provided the best inhibition of *G. boninense* with a PIRG of 94%, followed by THY at a concentration of 0.25 mg/mL with an inhibition of 87.13% (Ganapathy et al., 2021). Humic acid (HA) has been reported to increase plant membrane permeability (Lotfi et al., 2018). Similar to function of HA, but in different organisms, Gallic acid (GA) and Thymol (THY) have been reported to increase *G. boninense* membrane permeability and cause the depletion of components essential to fungal survival (Ganapathy et al., 2021). In the future, GA and THY had the potential to be used as a green control strategy and further developed as a natural antifungal agent to suppress *G. boninense*.

### 10.9 Innovative genetic tools

Genetic modification has been applied to confer resistance to oil palm against BSR infection. In 2020, oil palm embryogenic calli that co-bombarded with a construct carrying the *AGLU1* and *RCH10* genes have been reported to exhibited resistance to *G. boninense*. The results demonstrated that *G. boninense* resistance oil palm could be produced through biolistic bombardment (Hanin et al., 2020).

A cutting-edge CRISPR/Cas9 has been applied to introduce the genes in the oil palm calli (OPC). When delivered into oil palm callus, CRISPR/Cas9 constructs carrying sgRNA with targeted isoflavone reductase (IFR) and methallotioneine like protein (MT) genes produced mutations in both targeted DNA areas, altering the sequences of the amino acid residues (Budiani et al., 2018). A method like this might open up new possibilities for research into the defence genes responsible for disease resistance in oil palm. In 2021, effective and efficient CRISPR/Cas9 gene editing in oil palm have been achieved through maximising the choice of efficient gRNA and DNA delivery techniques. The genetic enhancement of the oil palm will be made possible by this newly developed technique (Yeap et al., 2021).

## 11 Challenges and future prospects

Control strategies is always the priority to be considered of to curb or control *G. boninense*. Albeit many control methods have been showed positive results in the *in-vitro* tests, but none of the methods can totally eradicate *G. boninense* in the field.

Diseased oil palm stumps are the tissues that are most contagious when *G. boninense* inoculum is present. To keep the plantation free of sources of pathogens, it is vital to remove unhealthy tissues (Chong et al., 2017b). This has led to a great deal of interest in the use of white-rot fungi (WRF), an environmentally friendly and biodegradable *Ganoderma* management strategy, to destroy the diseased stumps. According to Naidu et al. (2017), *Pycnoporus sanguineus* FBR and *Grammothele fuligo* ST2 are the strongest biodegraders of wood blocks that have been colonized by *G. boninense*. The findings have provided novel insights on the potential of the WRF to eradicate *Ganoderma* inoculums in the future.

Gene technology has been thought of as a potential improvement for the oil palm. The Malaysian Palm Oil Board (MPOB) and their American collaborators have sequenced and published the oil-palm genomes of the South American oil-palm *E. oleifera* and the African oil-palm *E. guineensis* to aid in the improvement study of oil palm (Singh et al., 2013). Through gene technology, genetically modified (GM) oil palm with traits such as oleic acid for healthy low unsaturated oil and high PHBV (polyhydroxybutyrate-co-valerate) for bioplastics production have been produced in the laboratory and biosafety containment houses (Yunus et al., 2008; Nurfahisza et al., 2014). The studies have proved that gene technology can improve the oil palm. However, GM oil palm faced technical and commercial problems, and public sensitivity (Murphy, 2017; Soh, 2018). Even if these obstacles in the way of technique and commerce can be addressed, public aversion to GM crops, which has existed in various regions of the world since 1999, means that new transgenic cultivars continue to encounter difficulties. Up until the present, this opposition has been particularly strong in Europe. As a result, many local regions in the EU implemented legal restrictions on the production of all GM crops in late 2015, even varieties that had received European Commission approval (Nelson, 2015).

In present era, the controversy of GM crops has been viewed as being resolved by the genome editing (GE) approach, which permits targeted *in situ* DNA change without or temporarily using transgenes (Mao et al., 2013). Integration of GE with tissue-culture-free methods would avoid the crops being labelled as genetically modified organism (GMO) (Hickey et al., 2019). The ability to do single or multiplex edits has been widely established (Zhang et al., 2016); this capability can now be put into practise using the following tissue-culture-free methods. For an instance, Clustered, regularly interspaced short palindromic repeat/CRISPR-associated protein (CRISPR/Cas9) ribonucleoproteins (RNPs) complexes can be used for GE. CRISPR-based GE enables precise target modifications in the DNA of an organism (Adli, 2018). This has been done for a variety of species, including potato, wheat (Liang et al., 2017), and maize (Andersson et al., 2018). Target tissues have included protoplasts or immature embryos; ideally, this technique would be enhanced for mature seeds or sprouting seedlings (Hamada et al., 2017). Current GMO rules in some countries may be addressed by CRISPR/Cas9 RNPs (Kanchiswamy, 2016; Lu et al., 2017); nevertheless, this technology can also be used to control oil palm diseases and pests (Khan et al., 2022).

## 12 Conclusion

**Figure 2:**
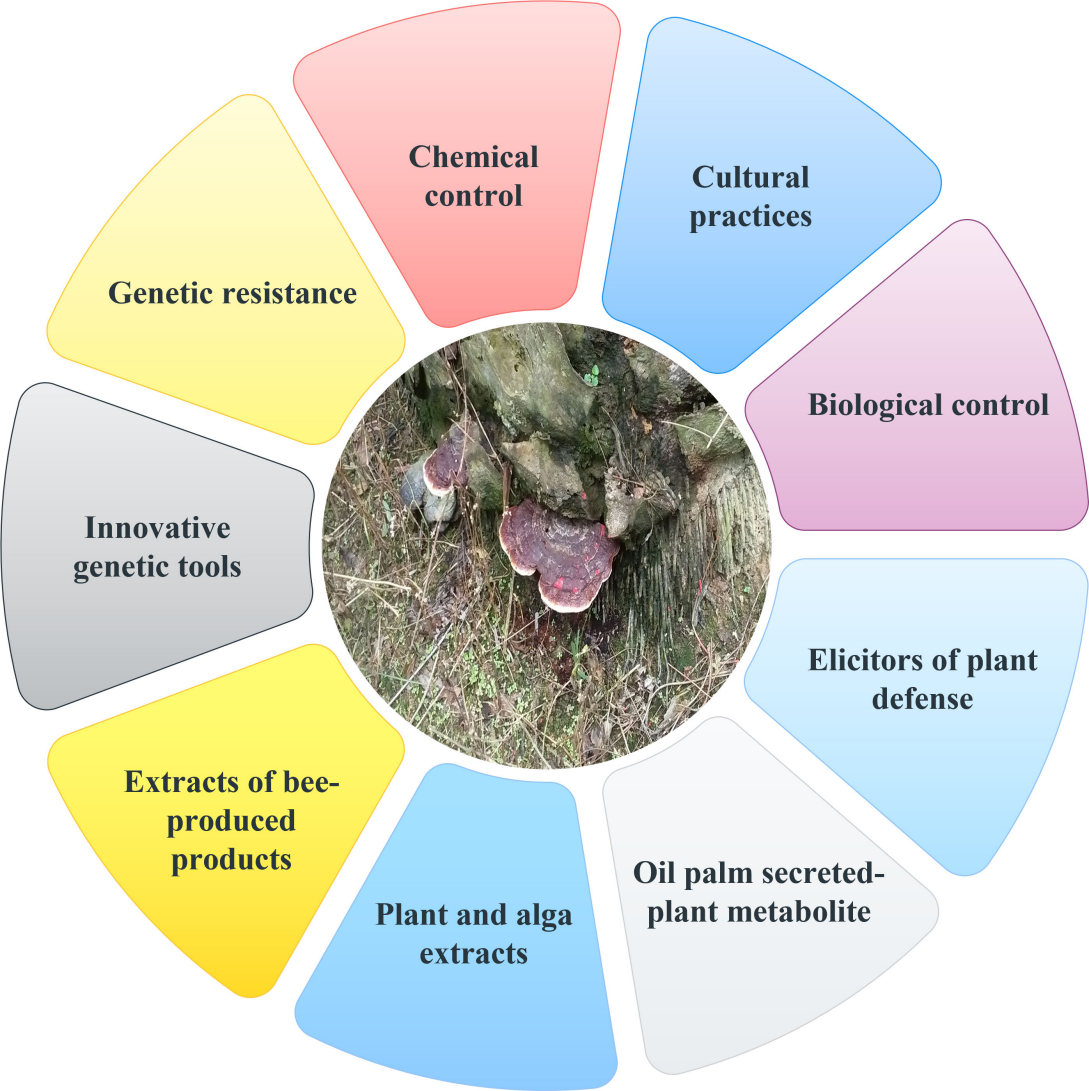
Current and potential management strategies of *Ganoderma boninense*.

Since the interactions between *G. boninense* and oil palm are so complicated, it is crucial to fully comprehend them in order to improve disease control measures. As there is no single effective control approach that can stop the spread of this disease, integrated disease management is necessary to manage BSR. Chemical fungicides, cultural practices, biological techniques, and fertilizer application are all examples of current management approaches. All current and potential management strategies are summarised in **Figure 2**. Reduced BSR incidence following replanting is the goal of BSR integrated disease management. Since the impact of this soilborne fungus lies in both the losses it causes and the effects it has on the environment as a result of unsustainable management practices, it is crucial to handle BSR brought on by *G. boninense* responsibly.

## Author contributions

Conceptualization, YK and KC; Writing-original draft preparation, YK; Writing-Review & Editing, KC; Supervision, KC. Both authors contributed to the article and approved the submitted version.
