# Corrigendum: *Ganoderma boninense*: general characteristics of pathogenicity and methods of control

**DOI:** 10.3389/fpls.2024.1360323

**Published:** 2024-01-24

**Authors:** Ying Wei Khoo, Khim Phin Chong

**Affiliations:** ^1^ State Key Laboratory for Biology of Plant Diseases and Insect Pests, Institute of Plant Protection, Chinese Academy of Agricultural Sciences, Beijing, China; ^2^ Faculty of Science and Natural Resources, Universiti Malaysia Sabah, Kota Kinabalu, Sabah, Malaysia

**Keywords:** control, diagnostics, *Ganoderma boninense*, oil palm, pathogenicity

In the published article, there was an error in affiliation 1. Instead of “Faculty of Science and Natural Resources, Universiti Malaysia Sabah, Sabah, Kota Kinabalu, Malaysia”, it should be “State Key Laboratory for Biology of Plant Diseases and Insect Pests, Institute of Plant Protection, Chinese Academy of Agricultural Sciences, Beijing, China”.

In the published article, there was an error in affiliation 2. Instead of “State Key Laboratory for Biology of Plant Diseases and Insect Pests, Institute of Plant Protection, Chinese Academy of Agricultural Sciences, Beijing, China”, it should be “Faculty of Science and Natural Resources, Universiti Malaysia Sabah, Kota Kinabalu, Sabah, Malaysia”.

The authors apologize for this error and state that this does not change the scientific conclusions of the article in any way. The original article has been updated.

